# CD11b maintains West Nile virus replication through modulation of immune response in human neuroblastoma cells

**DOI:** 10.1186/s12985-024-02427-6

**Published:** 2024-07-14

**Authors:** Yan-Gang Liu, Hao-Ran Peng, Rui-Wen Ren, Ping Zhao, Lan-Juan Zhao

**Affiliations:** 1 Department of Microbiology, Shanghai Key Laboratory of Medical Biodefense, Faculty of Naval Medicine, Naval Medical University, 800 Xiang-Yin Road, Shanghai, 200433 China; 2 Center for Disease Control and Prevention of Southern Theater Command, Guangzhou, China

**Keywords:** West Nile virus, CD11b, TNF-α, IFN

## Abstract

**Background:**

West Nile virus (WNV) is a rapidly spreading mosquito-borne virus accounted for neuroinvasive diseases. An insight into WNV-host factors interaction is necessary for development of therapeutic approaches against WNV infection. CD11b has key biological functions and been identified as a therapeutic target for several human diseases. The purpose of this study was to determine whether CD11b was implicated in WNV infection.

**Methods:**

SH-SY5Y cells with and without MEK1/2 inhibitor U0126 or AKT inhibitor MK-2206 treatment were infected with WNV. *CD11b* mRNA levels were assessed by real-time PCR. WNV replication and expression of stress (ATF6 and CHOP), pro-inflammatory (TNF-α), and antiviral (IFN-α, IFN-β, and IFN-γ) factors were evaluated in WNV-infected SH-SY5Y cells with *CD11b* siRNA transfection. Cell viability was determined by MTS assay.

**Results:**

*CD11b* mRNA expression was remarkably up-regulated by WNV in a time-dependent manner. U0126 but not MK-2206 treatment reduced the CD11b induction by WNV. CD11b knockdown significantly decreased WNV replication and protected the infected cells. CD11b knockdown markedly increased *TNF-α*, *IFN-α*, *IFN-β*, and *IFN-γ* mRNA expression induced by WNV. *ATF6* mRNA expression was reduced upon CD11b knockdown following WNV infection.

**Conclusion:**

These results demonstrate that CD11b is involved in maintaining WNV replication and modulating inflammatory as well as antiviral immune response, highlighting the potential of CD11b as a target for therapeutics for WNV infection.

**Supplementary Information:**

The online version contains supplementary material available at 10.1186/s12985-024-02427-6.

## Introduction

West Nile virus (WNV) belongs to the *Orthoflavivirus* genus within the Flaviviridae family and is an enveloped positive sense single-stranded RNA virus [1]. WNV, a mosquito-transmitted neurotropic virus, is one of the leading causes of zoonotic viral encephalitis in the world [2]. Birds, horses, and humans are all susceptible to WNV infection, leading to WNV maintenance in nature due to a large variety of hosts. In humans, WNV acute infection causes various clinical manifestations ranging from mild flu symptom to severe neuroinvasive consequence. Apart from the neuroinvasive diseases including meningitis, encephalitis, and flaccid paralysis, WNV infection may induce lesions in heart, kidneys, and eyes [3]. Recently, WNV has dramatically expanded its geographical range covering Africa, the Americas, Europe, and Asia [4]. The worldwide spread of WNV, combined with the lack of specific antiviral drugs and licensed human vaccines, poses an urgent need to identify the key processes implicated in WNV infection and pathology.

Upon WNV infection, host innate immune response is the first line of defense by controlling virus replication, limiting pathology, and inducing protective immunity, characterized by production of type I interferon (IFN) and interleukin-1β (IL-1β) as well as expression of antiviral effector genes [5]. Most studies have focused on WNV-immune response interaction that determines the pathogenesis and outcome of WNV infection. In human cases and rodent models, WNV infection causes neurological damage through induction of IL-1β, IFN-γ, and α-synuclein [6]. Type I IFN displays a dominant role in protection against lethal WNV infection by limiting WNV replication, spread, and pathology [7]. In patients with WNV fever and WNV neuroinvasive diseases, increased concentrations of the key cytokines involved in innate and early acute phase response (IL-6) and Th1 type immune response (IFN-γ) are found in the cerebrospinal fluid while the pro-inflammatory cytokine tumor necrosis factor α (TNF-α) appears to be concentrated mainly in the serum [8]. Further insight into how WNV interacts with host factors is necessary for development of therapeutic approaches against WNV infection.

The β_2_ integrin family is composed of four members including CD11a (αL), CD11b (αM), CD11c (αX), and CD11d (αD) subunits paired with the β_2_ subunit CD18, respectively. As adhesion and signaling molecules, β_2_ integrins play important roles in regulating innate immune cell functions [9]. CD11b, the α subunit of integrin CD11b/CD18 (also known as α_M_β_2_, macrophage-1 antigen, and complement receptor 3), is highly expressed on the surface of innate immune cells and has key biological functions in immune cells and some signaling pathways [10]. Emerging evidence suggests that variations at the human *ITGAM* gene encoding the CD11b are strongly associated with susceptibility to systemic lupus erythematosus and lupus nephritis [10, 11]. Moreover, CD11b/CD18 has been regarded as a molecule with pro-inflammatory, anti-inflammatory, and immunoregulatory functions [12]. Thus, dysregulation of these functions may contribute to inflammation, immunity, and infection. Due to its multi-functions, CD11b has been implicated in the disease development and emerged as a potential target for therapy. For example, bone marrow-derived CD11b(+)Jag2(+) cells trigger onset of colorectal cancer progression through induction of epithelial-to-mesenchymal transition in tumor cells [13]. CD11b(+) myeloid cells promote liver metastasis by down-regulating angiopoietin-like 7 expression in cancer cells [14]. Involvement of CD11b in microglial nicotinamide adenine dinucleotide phosphate oxidase activation induced by α-synuclein provides a novel mechanistic insight for synucleinopathies [15]. Importantly, CD11b represents a susceptibility factor for autoimmunity and could be a target for future therapy [16]. CD11b is also identified as a novel therapeutic target for ovarian cancer [17]. Interaction between CD11b/CD18 and glycoprotein Ibα seems to distinguish thrombosis from hemostasis, supporting the possibility of new antithrombotic therapeutic target [18]. Studies have made progress in dissecting roles of CD11b in the development and progression of human diseases.

The mechanisms underlying WNV-mediated immunopathology remain to be investigated in depth. We focused on the interaction of WNV with host factors that influences viral pathogenesis and outcome of WNV infection. Based on the features of immune response elicited by WNV infection and the distinct biological functions of CD11b, we addressed whether CD11b was implicated in WNV infection and how the immune response influenced WNV infection. We present results that CD11b is involved in maintaining WNV replication and differentially regulating expression of activating transcription factor 6 (ATF6), cyclic AMP response element-binding transcription factor homologous protein (CHOP), TNF-α, and IFNs.

## Materials and methods

### Cells and viruses

Human neuroblastoma SH-SY5Y cells (ATCC CRL-2266) were used for virus infection. African green monkey kidney Vero cells (ATCC CCL-81) were used for virus propagation. Cells were cultured in Dulbecco’s modification of Eagle’s medium (Invitrogen, Carlsbad, USA) containing 10% fetal bovine serum, 1% L-glutamine, 1% non-essential amino acids, and 1% penicillin-streptomycin (complete medium) at 37 °C in a 5% CO_2_ atmosphere. WNV and tick-borne encephalitis virus (TBEV) working stocks were prepared and titrated by plaque assay as described [19, 20]. Experiments with virus infection were carried out in the Biological Safety Level 3 Laboratory in accordance with guidelines by the Committee on Safety of Biomedicine at Naval Medical University (Shanghai, China). Culture supernatants of Vero cells were collected as mock inoculum. Aliquots of viruses and the culture supernatants (mock inoculum) were stored at -80 °C until use.

### Virus infection

Confluent monolayer of SH-SY5Y cells in 35 mm culture dishes was incubated with WNV at a multiplicity of infection (MOI) of 2 or 0.1 MOI of TBEV for 1 h of adsorption at 37 °C. After removal of the unbound virus and wash with phosphate-buffered saline (PBS), the cells were grown in fresh complete medium for 12, 24, and 48 h, respectively. Cells were cultured for the corresponding time periods in complete medium containing the culture supernatants of Vero cells as a mock control. The time points were measured from the end of the 1 h adsorption.

### Kinase inhibitor treatment and WNV infection

SH-SY5Y cells in 12-well culture plates at a confluent monolayer were pretreated for 1 h at 37 °C with 10 µM of MEK1/2 inhibitor U0126 (Cell Signaling Technology, Beverly, USA) or 1 µM of AKT inhibitor MK-2206 (Selleck, Houston, USA), and washed twice with PBS followed by incubation for 1 h with 2 MOI of WNV. The cells were washed two times with PBS and maintained in fresh complete medium containing the U0126 or the MK-2206 for another 24 h. The U0126 and MK-2206 inhibitors were dissolved in dimethylsulfoxide (DMSO; Sigma, St.Louis, USA). As a dissolvent control, cells were treated with DMSO and then infected with WNV.

### siRNA transfection and WNV infection

SH-SY5Y cells were seeded in 24-well culture plates and reached appropriately 30-50% confluence. *CD11b* siRNA (Catalog number stB0001176A; Ribobio, Guangzhou, China) or Signal Silence Control siRNA (Catalog number 6568; Cell Signaling Technology) was transfected into cells at a final concentration of 100 nM using Lipofectamine 2000 (Invitrogen) according to the manufacturer’s instructions. At 48 h post transfection, the cells were incubated with 2 MOI of WNV for 1 h adsorption, washed with PBS, and subsequently cultured for 48 h.

### Viability assay

At 48 h post infection, SH-SY5Y cells with and without the siRNA transfection were assayed for cell viability using CellTiter 96^®^AQueous One Solution Cell Proliferation kit containing MTS (Promega, Madison, USA) following the manufacture’s instructions as described previously [19].

### RNA extraction, reverse transcription and real-time PCR

Total RNA was extracted from the cells using Trizol (Invitrogen). The extracted RNA was subsequently reverse transcribed into cDNA using moloney murine leukemia virus reverse transcriptase kit (Promega). For detection of expression of target genes, quantitative real-time PCR was run using SYBR Green PCR kit (Promega) on a Rotor-Gene 3000 Thermal Cycler (Corbett, Australia). The cycling conditions were 2 min at 95 °C, followed by 40 cycles of 10 s at 95 °C, 10 s at 55 °C, and 25 s at 72 °C. Expression values were normalized to glyceraldehyde-3-phosphate dehydrogenase (GAPDH) endogenous control. The relative expression levels were calculated by the threshold cycle (2^−ΔΔCT^) method. The primers used were listed in Supplementary Table S1. The primers for TBEV and GAPDH were described previously [19].

### Statistical analysis

Data are shown as means and standard deviation and representative of at least three experiments. Two-group comparisons were performed by Student’s t-test and multiple-group comparisons were performed by one-way ANOVA with Tukey’s post-hoc test (GraphPad Prism 8.0 software). Statistically significant differences are considered for ^*^P < 0.05, ^**^P < 0.01, ^***^P < 0.001, and ^****^P < 0.0001.

## Results

### WNV infection dynamically up-regulates *CD11b* mRNA expression

**Fig. 1:**
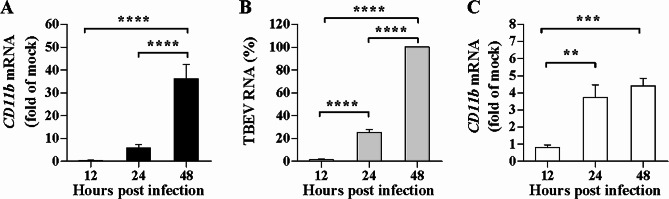
Kinetics of *CD11b* mRNA expression induced by WNV in human neuroblastoma cells. (**A**) SH-SY5Y cells were infected with WNV at an MOI of 2 and *CD11b* mRNA levels were quantified and shown as relative fold of the mRNA levels in the cells infected with WNV over the levels in the cells incubated with the culture supernatants of Vero cells (mock inoculum) at the indicated time points. (**B**) SH-SY5Y cells were infected with TBEV at an MOI of 0.1 for the indicated time periods. TBEV RNA levels were quantified and shown as relative percentages of the control at 48 h post infection. (**C**) *CD11b* mRNA levels were quantified and shown as relative fold of the mRNA levels in SH-SY5Y cells infected with 0.1 MOI of TBEV over the levels in the cells incubated with the mock inoculum at the indicated time points. One-way ANOVA with Tukey’s post-hoc test.^**^P < 0.01, ^***^P < 0.001, ^****^P < 0.0001, *n* = 3

Neuronal cell lines are commonly applied to determine neurovirulence of neurotropic viruses and regarded as experiment models in vitro for elucidation of the neuropathogenesis [21, 22]. The impact of WNV infection on CD11b expression was then analyzed in human neuronal cells. We found that WNV established a productive infection in SH-SY5Y cells [23]. Here *CD11b* mRNA levels were detected in the SH-SY5Y cells infected with WNV for the various time periods. Figure 1A showed that WNV infection potently up-regulated *CD11b* mRNA expression in the SH-SY5Y cells. In comparison with *CD11b* mRNA levels at 12 h post infection, the *CD11b* mRNA levels were obviously increased at 24 h and a significant increase in the levels was observed at 48 h post infection (*P* < 0.0001). Regulation of *CD11b *mRNA expression by TBEV, an important neurotropic arbovirus, was additionally evaluated in SH-SY5Y cells. As expected, efficient replication of TBEV was observable in the SH-SY5Y cells (Fig. 1B). TBEV RNA levels were significantly increased at 48 h post infection as compared with those at 12–24 h (*P* < 0.0001), suggesting a time-dependent increase in TBEV replication. *CD11b* mRNA expression was also enhanced in the TBEV-infected SH-SY5Y cells (Fig. 1C). The *CD11b* mRNA levels were significantly increased at 24 h (*P* < 0.01) or 48 h (*P* < 0.001) post infection as compared with those at 12 h. While WNV infection led to strong enhancement of CD11b expression. Thus, *CD11b* mRNA levels were markedly up-regulated by WNV infection in a time-dependent manner.

### U0126 and MK-2206 treatment impacts CD11b induction by WNV

**Fig. 2:**
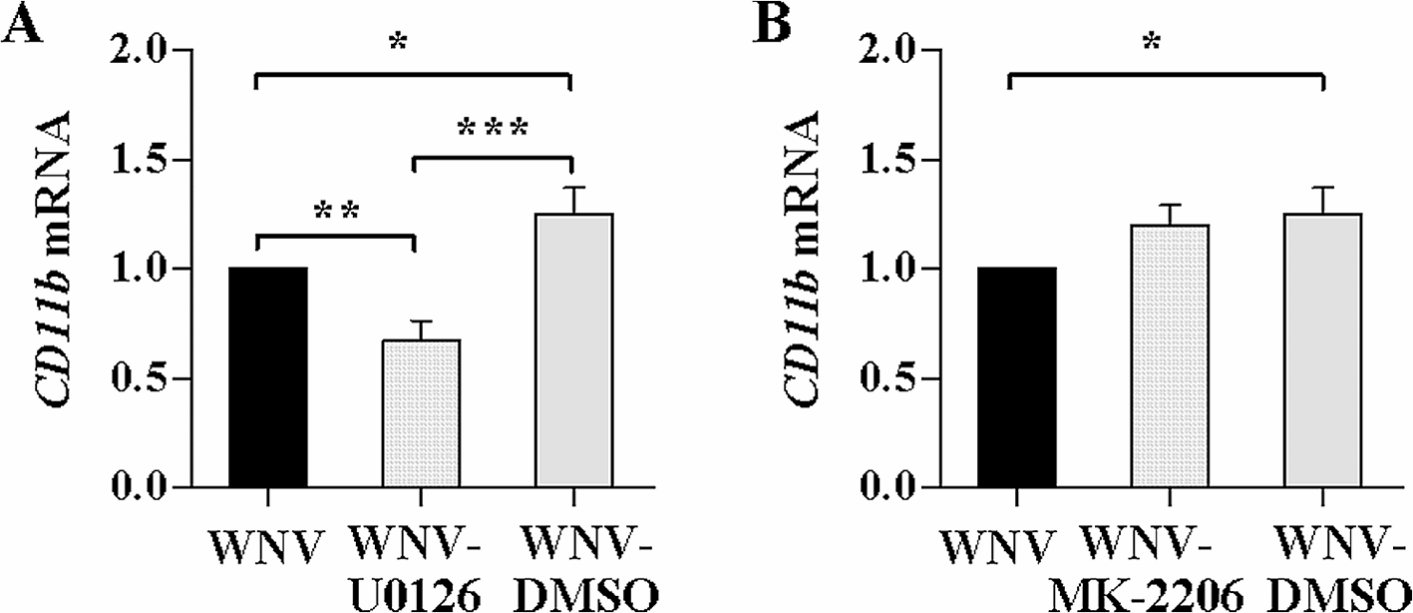
Effects of U0126 and MK-2206 on *CD11b* mRNA expression induced by WNV. SH-SY5Y cells were treated with U0126 (**A**) or MK-2206 (**B**) prior to WNV infection (MOI of 2). *CD11b* mRNA levels were quantified and shown as relative fold of the mRNA levels in the infected cells with the treatment over the levels in the cells without the treatment. One-way ANOVA with Tukey’s post-hoc test. ^*^P < 0.05, ^**^P < 0.01, ^***^P < 0.001, *n* = 3

CD11b takes part in several cellular signaling pathways, which is implicated in its biological functions [10]. Our results showed that kinase cascades of AKT (AKT-S6-4E-BP1) and ERK (MEK1/2-ERK-p90RSK) pathways were early signaling events involved in WNV infection and that the kinase inhibitors MK-2206 or U0126 played important roles in the WNV-mediated AKT and ERK activation as well as WNV replication in SH-SY5Y cells [23]. As described above, CD11b expression was markedly promoted in the SH-SY5Y cells infected with WNV. To explore whether the signaling events are related to the CD11b expression, AKT inhibitor MK-2206 or MEK1/2 inhibitor U0126 was used to treat SH-SY5Y cells prior to WNV infection. Figure 2A showed that *CD11b* mRNA levels were significantly decreased in the cells pretreated with 10 µM of U0126 as compared with WNV infection (*P* < 0.01). As a dissolvent control, the DMSO treatment increased *CD11b *mRNA expression (*P* < 0.05), which ruled out the inhibitory effect of DMSO. The *CD11b* mRNA levels were higher in the cells treated with the DMSO than those with the U0126 treatment (*P* < 0.001). Indeed, the U0126 treatment reduced the WNV-induced CD11b expression. There was no obvious difference in *CD11b* mRNA levels in the infected cells with and without 1 µM of MK-2206 treatment (Fig. 2B), implying MK-2206 treatment has no apparent impact on the induction of CD11b by WNV. These results suggest that ERK pathway may be involved in CD11b induction by WNV infection.

### CD11b knockdown decreases WNV replication

**Fig. 3:**
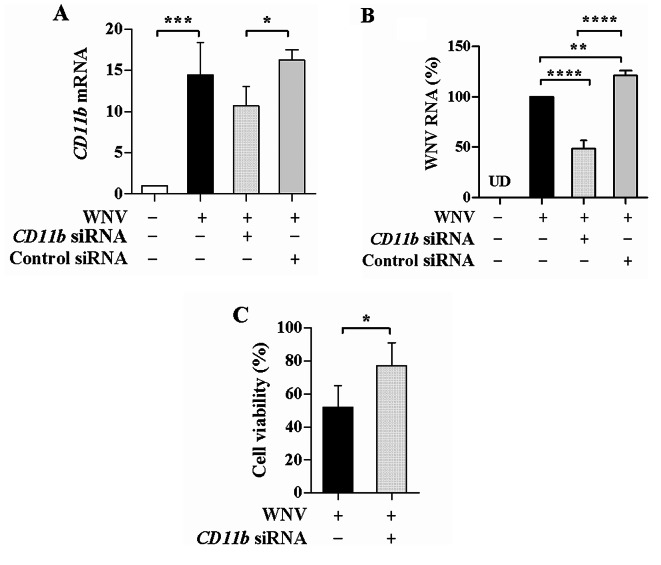
Influence of CD11b knockdown on WNV replication and cell viability. SH-SY5Y cells were transfected with *CD11b* siRNA or control siRNA. At 48 h post transfection, the cells were infected with WNV (MOI of 2) for 48 h. (**A**) *CD11b* mRNA levels were determined and shown as relative fold of the mRNA levels in the infected cells with and without the siRNA transfection over the levels in the uninfected and untransfected cells. (**B**) WNV RNA levels were determined and shown as relative percentages of the infected control. UD, undetectable. One-way ANOVA with Tukey’s post-hoc test. ^*^P < 0.05, ^**^P < 0.01, ^***^P < 0.001, ^****^P < 0.0001, *n* = 3. (**C**) Cell viability was determined upon the *CD11b* siRNA transfection and shown as relative percentages of the uninfected and untransfected control. Student’s t-test. ^*^P < 0.05, *n* = 4

We found that the *CD11b* siRNA transfection led to nearly a 60% reduction in *CD11b* mRNA levels in SH-SY5Y cells. Influence of CD11b knockdown on WNV replication was assessed in WNV-infected SH-SY5Y cells with the siRNA transfection. Figure 3A showed that WNV infection increased *CD11b* mRNA levels as compared with the uninfected control (*P* < 0.001). *CD11b* mRNA levels were significantly reduced in the cells transfected with the *CD11b* siRNA compared with the control siRNA (*P* < 0.05), indicating CD11b knockdown in the WNV-infected cells. Due to the marked up-regulation of CD11b by WNV, the *CD11b* mRNA levels were insufficient reduced based on the *CD11b* siRNA transfection. At the same time, WNV replication was measured in the cells with and without the siRNA transfection. WNV replication was obviously inhibited due to the CD11b knockdown. WNV RNA levels were potently reduced in the cells transfected with the *CD11b* siRNA compared with the untransfected control or the control siRNA (*P* < 0.0001, Fig. 3B). WNV RNA levels were higher in the cells with the control siRNA transfection than those in the untransfected cells (*P* < 0.01), ruling out inhibitory effects of the siRNA transfection. Moreover, the viability of WNV-infected cells was lower than that of the cells with the *CD11b* siRNA transfection (Fig. 3C), which was consistent with the reduced WNV replication upon CD11b knockdown. The results showed that CD11b knockdown decreased WNV replication and protected the infected cells.

### CD11b knockdown differentially influences mRNA expression of stress and pro-inflammatory factors induced by WNV

**Fig. 4:**
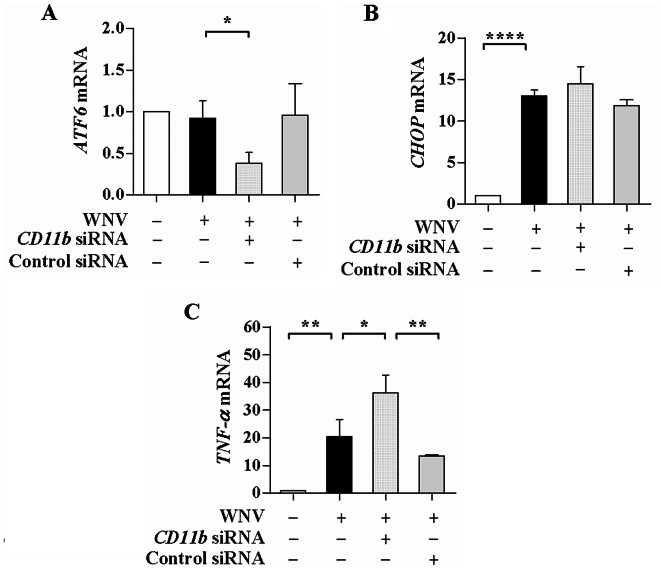
Influence of CD11b knockdown on mRNA expression of stress and inflammatory factors induced by WNV. SH-SY5Y cells were transfected with *CD11b* siRNA or control siRNA. At 48 h post transfection, the cells were infected with WNV (MOI of 2) for 48 h. The mRNA levels of *ATF6* (**A**), *CHOP* (**B**), and *TNF-α* (**C**) were analyzed and shown as relative fold of the mRNA levels in the infected cells with and without the siRNA transfection over the levels in the uninfected and untransfected cells. One-way ANOVA with Tukey’s post-hoc test. ^*^P < 0.05, ^**^P < 0.01, ^****^P < 0.0001, *n* = 3

WNV infection elicits strong inflammation response. CD11b has inflammatory and immune regulatory functions. It is thus interesting to define whether CD11b is responsible for the induction of inflammation response by WNV. The mRNA expression of stress and pro-inflammatory factors was analyzed in WNV-infected SH-SY5Y cells with CD11b knockdown. There were no obvious changes in *ATF6* mRNA levels in the cells with and without WNV infection, whereas the *ATF6* mRNA levels were significantly reduced in the cells transfected with the *CD11b* siRNA (*P* < 0.05, Fig. 4A). Figure 4B showed that WNV infection significantly increased the *CHOP* mRNA levels (*P* < 0.0001). The *CHOP* mRNA levels were not obviously affected in the cells transfected with the *CD11b* siRNA. The control siRNA transfection had no apparent impact on the mRNA expression of *ATF6* and *CHOP*. The stress factors ATF6 and CHOP were differently regulated by WNV infection and that CD11b knockdown decreased the *ATF6 *mRNA expression without affecting the *CHOP *mRNA expression. WNV infection distinctly enhanced the pro-inflammatory cytokine expression. As shown in Fig. 4C, *TNF-α* mRNA levels were significantly increased in the WNV-infected cells (*P* < 0.01). Interestingly, the mRNA levels of *TNF-α* were markedly increased in the *CD11b* siRNA-transfected cells (*P* < 0.05). The control siRNA transfection showed no significant effects on the *TNF-α* mRNA expression in the infected cells. These results indicated that CD11b participated in regulation of ATF6 and TNF-α by WNV infection.

### CD11b knockdown increases *IFN* mRNA expression induced by WNV

**Fig. 5:**
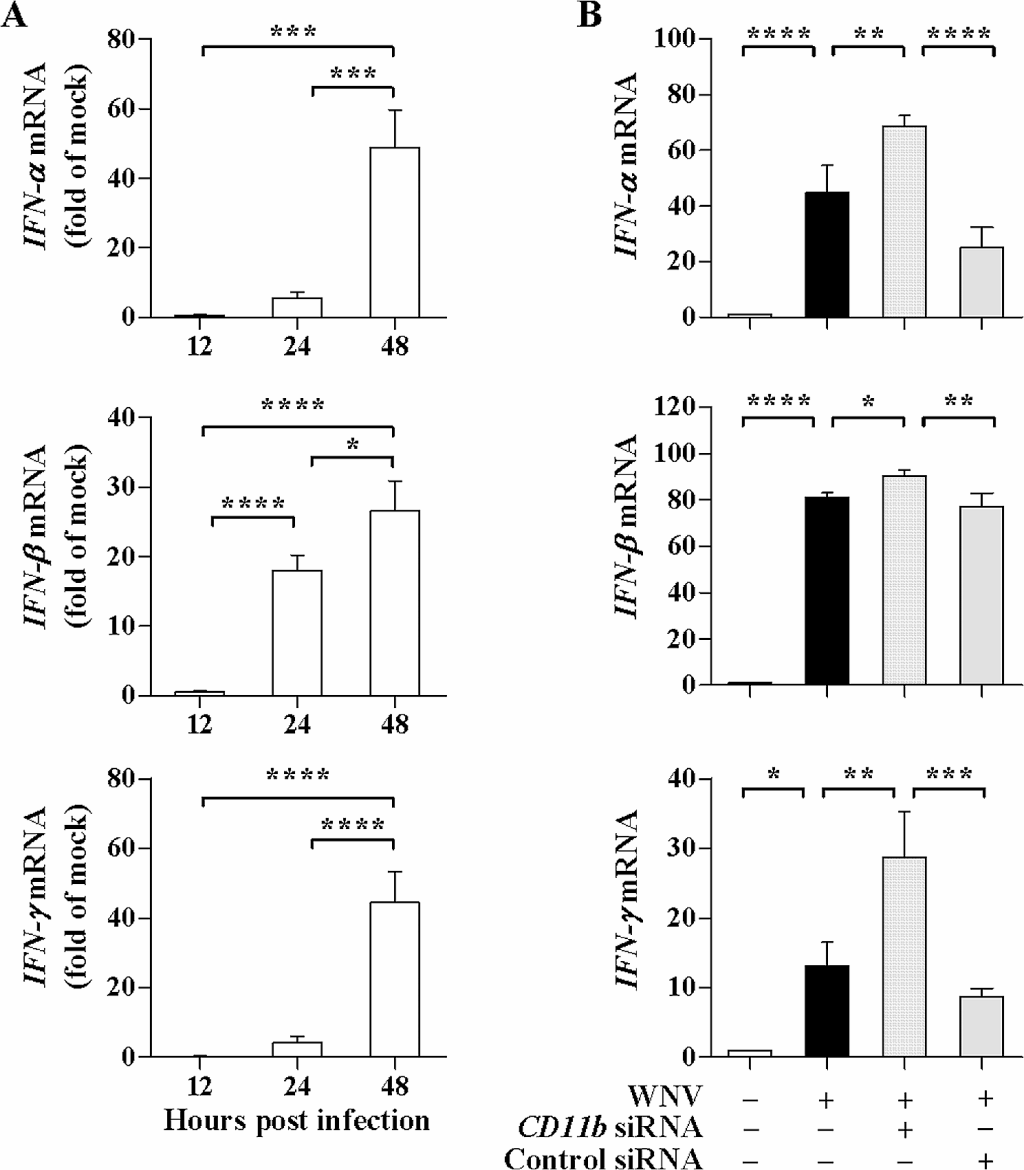
Influence of CD11b knockdown on *IFN* mRNA expression induced by WNV. (**A**) SH-SY5Y cells were infected with 2 MOI of WNV and mRNA levels of *IFN-α*, *IFN-β*, and *IFN-γ* were quantified and shown as relative fold of the mRNA levels in the cells infected with WNV over the levels in the cells incubated with the culture supernatants of Vero cells (mock inoculum) at the indicated time points. (**B**) SH-SY5Y cells were transfected with *CD11b *siRNA or control siRNA. At 48 h post transfection, the cells were infected with WNV (MOI of 2) for 48 h. The mRNA levels of *IFN-α*, *IFN-β*, and *IFN-γ* were analyzed and shown as relative fold of the mRNA levels in the infected cells with and without the siRNA transfection over the levels in the uninfected and untransfected cells. One-way ANOVA with Tukey’s post-hoc test. ^*^P < 0.05, ^**^P < 0.01, ^***^P < 0.001, ^****^P < 0.0001, *n* = 3

Furthermore, influence of CD11b knockdown on antiviral cytokine induction by WNV was analyzed. During WNV infection, kinetics of *IFN* mRNA expression was first assessed in SH-SY5Y cells. Figure 5A showed that WNV infection potently up-regulated mRNA expression of *IFN-α*, *IFN-β*, and *IFN-γ*. At 12 h post infection, the expression of *IFN-α*, *IFN-β*, and *IFN-γ* was detectable in the SH-SY5Y cells. A strong defense response was elicited by WNV at 48 h post infection, as evidenced by significant enhancement of *IFN-α*, *IFN-β*, and *IFN-γ* mRNA expression. The *IFN* mRNA levels were much higher at 48 h post infection than those at 12 h (for *IFN-α P* < 0.001; for *IFN-β* or *IFN-γ P* < 0.0001) as well as those at 24 h (for *IFN-α P* < 0.001; for *IFN-β P* < 0.05; for *IFN-γ P* < 0.0001). Thus, WNV infection induced expression of IFN-α, IFN-β, and IFN-γ.

Next, the *IFN* mRNA levels were measured upon CD11b knockdown. A robust increase in mRNA levels of *IFN-α*, *IFN-β*, and *IFN-γ* was detectable in SH-SY5Y cells infected with WNV (Fig. 5B). The mRNA levels of *IFN-α*, *IFN-β*, and *IFN-γ* were all significantly increased in the *CD11b* siRNA-transfected cells as compared with those in the WNV-infected cells (for *IFN-α* or *IFN-γ P* < 0.01; for *IFN-β P* < 0.05). In addition, the control siRNA transfection led to a significant decrease in the mRNA levels, suggesting CD11b knockdown is responsible for the increased mRNA levels of *IFN-α*, *IFN-β*, and *IFN-γ*. Therefore, CD11b was involved in the IFN-α, IFN-β, and IFN-γ induction by WNV in SH-SY5Y cells.

## Discussion

WNV is a widespread arbovirus that is responsible for severe neuroinvasive diseases in humans. Since innate immune response plays a major role in the control of WNV infection, modulation of the immune response is thereby a key process in determining the disease outcome. The lack of effective therapeutics and approved vaccines has been a significant challenge for combating WNV infection. Due to the ability to modulate cell adhesion, migration, and phagocytosis, CD11b is regarded as a target for the autoimmune disease therapy. We sought to elucidate roles of CD11b in WNV neuropathogenesis, which may offer a novel insight for the development of therapeutics for WNV infection.

WNV could attack the central nervous system and cause encephalitis characterized by acute neuroinflammation in the brain. To define how the response is programmed and regulated during WNV infection, a human neuroblastoma cell line SH-SY5Y was used as an experiment model for dissecting the interaction of WNV with CD11b in the present study. We found that WNV infection markedly promoted the *CD11b* mRNA expression and such promotion was induced by WNV in a time-dependent manner. Influence of TBEV, the other major pathogen of arboviral neuroinvasive diseases, on CD11b was also evaluated. The *CD11b* mRNA expression was obviously enhanced in the TBEV-infected SH-SY5Y cells, which was consistent with the increased TBEV RNA replication. In comparison with TBEV, WNV displayed strong promotion of CD11b expression, implying distinct regulation of CD11b and differential roles of CD11b during virus infection. In supporting our findings, previous studies showed that a threefold increase in CD45(int) CD11b(+) microglia was triggered in lethal WNV mice model and that pathogenic Ly6C(high) CD11b(+) monocytes were the major infiltrating subset in the central nervous system of WNV-infected mice [24, 25]. Moreover, cellular processes involved in the CD11b regulation by WNV were assessed by using the inhibitor for AKT or MEK1/2. Our results suggest that ERK pathway is engaged in the modulation of CD11b by WNV, identifying an association of WNV-CD11b-ERK. On the other hand, whether CD11b expression affected WNV replication was assessed by using the *CD11b* siRNA. Of interest, CD11b knockdown led to the decreased WNV replication accompanied with the increased survival of infected cells. Therefore, up-regulation of CD11b expression induced by WNV infection may facilitate viral infection and replication.

Many flaviviruses manipulate multiple signaling pathways, including autophagic, innate immune, and stress response, to benefit viral replication. ATF6, a major member of unfolded protein response pathways, is activated by WNV, leading to CHOP induction [26]. ATF6 signaling is required for efficient WNV replication by promoting cell survival and inhibiting innate immune response [27]. The experiments were performed to test the biological significance of CD11b during WNV infection. We observed that WNV infection enhanced the *CHOP* mRNA expression without enhancement of the *ATF6* mRNA expression. However, CD11b knockdown decreased the *ATF6* expression without affecting the *CHOP* expression, implying that CD11b may play differential roles in the stress response during WNV infection. Inflammation is a major contributor to WNV encephalitis morbidity. We evaluated changes in the mRNA levels of key pro-inflammatory cytokine TNF-α in WNV-infected cells. As expected, WNV infection significantly increased *TNF-α* mRNA expression, which is consistent with previous findings regarding the induction of TNF-α by WNV in human neuroblastoma SK-N-SH cells, primary human fetal brain neural stem cell system, immune cells of patients with symptomatic WNV infection, and neurons from murine model of WNV encephalitis [28–31]. CD11b knockdown was responsible for the promotion of *TNF-α* expression induced by WNV. Our results suggested that CD11b took part in the WNV-mediated inflammation response, providing a possibility for the development of therapeutic strategies to reduce inflammatory symptoms associated with WNV. How WNV infection elicits stress and inflammation response by targeting CD11b is largely unknown. Further research into the regulation of cellular processes is needed.

The IFN defense has been extensively studied because of protection against lethal WNV infection [7]. For example, protective roles for IFN-α and IFN-γ against WNV are shown in mice [32]. In primary human dermal fibroblasts, WNV replication is constrained late in infection by an IFN-β-dependent reduction [33]. WNV infection induces secretion of type I IFN by human dendritic cells [34]. Early IFN-β induction regulates WNV replication in human glioblastoma cells, whereas delayed IFN-β induction results in efficient virus replication in neuroblastoma SK-N-SH cells [35]. Our results demonstrated that WNV infection elicited strong antiviral response characterized by profoundly increased expression of IFN-α, IFN-β, and IFN-γ. Upon CD11b knockdown, the mRNA levels of *IFN-α*, *IFN-β*, and *IFN-γ* were all significantly increased in the infected cells, suggesting involvement of CD11b in such induction. Moreover, the increased levels of IFNs may contribute to the decreased WNV replication. We propose that, by manipulating CD11b, WNV utilizes the cellular response to CD11b up-regulation to facilitate viral infection and replication. A greater understanding of the mechanisms underlying WNV neuropathogenicity is important for the development of effective antiviral therapy. CD11b may represent a key host factor implicated in cellular processes that control the outcome of and immunity to WNV infection.

Collectively, our results demonstrate the importance of CD11b in maintaining WNV replication and modulating inflammatory as well as antiviral immune response in human neuroblastoma SH-SY5Y cells. Further studies will be needed to comprehensively explore the association between WNV infection and CD11b government based on experiment animal models and various strains of WNV.

### Electronic supplementary material

Below is the link to the electronic supplementary material.

Supplementary Material 1

## Data Availability

No datasets were generated or analysed during the current study.
