# Supplementary material for: CD11b maintains West Nile virus replication through modulation of immune response in human neuroblastoma cells
Source: Virol J. 2024 Jul 14;21:158. doi: 10.1186/s12985-024-02427-6 (PMC11247799; doi:10.1186/s12985-024-02427-6)
Supplement: Supplementary file 1 — Supplementary Material 1 [file 12985_2024_2427_MOESM1_ESM.doc]

**Table S1. Primer pairs for real-time PCR**

| **Gene** | **Sense primer（5’-3’）** | **Antisense primer（5’-3’）** |
| --- | --- | --- |
| *ATF6* | CTTTTAGCCCGGGACTCTTT | TCAGCAAAGAGAGCAGAATCC |
| *CD11b* | AAGATGCCCACTGAGGAATG | TCTGGAAAGGGAGACTTTTCAC |
| *CHOP* | AGCTGGAACCTGAGGAGAGA | TGGATCAGTCTGGAAAAGCA |
| *IFN-α* | TAGGCTCACCCATTTCAACCAG | CAGGAGGGCCACCAGTAAAG |
| *IFN-β* | GACATCCCTGAGGAGATTAAGCA | CAACAATAGTCTCATTCCAGCCA |
| *IFN-γ* | ACTAGGCAGCCAACCTAAGCAAGA | TGGAAGCACCAGGCATGAAATCTC |
| *TNF-α* | TCCGTGAAAACGGAGCTGAA | AGGCTCAGCAATGAGTGACA |
| *WNV* | GAGTCCAAGAAGTCAGAGGGTACA | CCACTCTTCATGGTGACAATGTTCC |
